# Preparation of Compositional Gradient Polymeric Films Based on Gradient Mesh Template

**DOI:** 10.3390/polym10060677

**Published:** 2018-06-18

**Authors:** Honglei Teng, Jing Li, Zhaosheng Hou, Xilu Yan, Linru Han, Jing Xu, Tianduo Li

**Affiliations:** 1 Shandong Provincial Key Laboratory of Molecular Engineering, School of Chemistry and Pharmaceutical Engineering, Qilu University of Technology (Shandong Academy of Sciences), Jinan 250353, China; Tenghonglei1122@163.com (H.T.); lijing@163.com (J.L.); yanxilu99115@163.com (X.Y.); hanrulin99522@163.com (L.H.); 2 College of Chemistry, Chemical Engineering and Materials Science, Shandong Normal University, Jinan 250100, China; houzs@sdnu.edu.cn

**Keywords:** compositional gradient, gradient mesh template, filling method, hydrophilic/hydrophobic, water vapor permeability

## Abstract

In this paper, a template-filling method was found to prepare composition gradient gelatin films by incorporating α-[3-(2,3-epoxypropoxy) propyl]-ω-butyl-polydimethylsiloxane (PDMS–E) grafted gelatin (PGG) into a gradient gelatin mesh template. The method can be used to prepare other composition gradient biopolymer films. Gradient mesh template prepared by the methacrylic anhydride cross-linked gelatin under temperature gradient field. The porosity of the template decreased from 89 to 35% which was accompanied by decrease in average pore size from 160 to 50 µm. Colloidal particles about 0.9~10 µm were formed from PGG after adding them to a mixed solvent system of 9:1 (*v*/*v*) of ethanol/water, which were filled in the mesh template under vacuum (0.06 MPa). A gradient film was obtained after drying at room temperature for 48 h. The results of scanning electron microscope-energy dispersive X-ray combined with freezing microtome and Fourier transform infrared spectroscopy suggested that the distribution of the Si element along the thickness showed a typical gradient pattern, which led to hydrophilic/hydrophobic continuous changing along the thickness of film. The water vapor permeability, thermal gravimetric analysis, differential scanning calorimetry and dynamic mechanical tensile results show that the gradient films had excellent water vapor permeability and flexibility, and hence could be used as biomimetic materials and leather finishing agents.

## 1. Introduction

Gelatin, a proteinaceous material obtained by the hydrolytic degradation of naturally occurring collagen, shows the main advantages of natural macromolecules but no synthetic equivalent which is usually available in biomaterials [1,2,3,4]. However, applications of gelatin are significantly limited, due to its poor mechanical properties and water-resistant [5,6]. Reports suggest that both, the chemical and physical properties of gelatin, can be tuned by introducing a mono epoxy terminated polydimethylsiloxane (PDMS-E) macromonomer [7,8] which is a polymer that exhibits special low surface free energy, super-hydrophobic, low glass transition temperature, biocompatibility, excellent gas permeability and exceptional elasticity when lightly cross–linked [9,10]. Our previous studies focus mainly on the effect of aggregation and interactions between anionic surfactants and gelatin on the grafting density of gelatin modified with PDMS-E [7,8]. Furthermore, the effect of electrostatic and hydrophobic interactions on the solid-state structure of PDMS-E grafted gelatin (PGG) was studied. Based on these studies, we expected to overcome the inherent water-resistance and mechanical properties defects of gelatin films utilizing the favorable properties of both gelatin and PDMS segments, and simultaneously provided the excellent water vapor permeability and flexibility for gelatin film. Composition gradient film of PGG may be realizing the wish.

Functionally graded materials (FGMs) in which the compositions and/or functionalities are continuously and spatially varied along one or more dimensions can fabric the expected materials, such as a material without any concentration of mechanical stress, mechanical properties controlled by spatially. Due to the concentration gradient of the polymers, many important properties of the films such as wettability, mechanical/optical/thermal properties, density of functional groups and cell adhesion as a function of the elastic modulus can be finely tuned. The gradient films can be prepared by many methods, which include dissolution and diffusion methods [11], temperature gradient field method [12], as interpenetrating polymer networks [13], by extrusion [14,15], microfluidic techniques [16,17], electrochemical technique [18], centrifugation [19], and so on [20,21,22]. Till date, FGMs have been potentially used in various fields such as medicine [23,24,25,26,27,28], biology [29,30,31,32], and optics [5,33,34,35]. In this regards, preparation of gradient films by incorporating PDMS components along the thickness would be an effective way to fabric the favorable properties of both gelatin and PDMS segments.

However, as a polyelectrolyte, complicated non-covalent interactions exist among gelatin molecules, which are known to be sensitive to a variety of factors including concentration, solvent, pH, temperature and additives such as surfactant [30,31]. In addition, the difference of compatibility between PDMS and gelatin will induce aggregation of PDMS-E grafted gelatin in aqueous solution or organic solvent. For all the above reasons, existing preparation methods for preparation FGMs are difficult to directly apply in producing the graded gelatin films.

Karageorgiou et al. fabricated a pore size gradient across a slab made of silk fibroin. Salt-leaching combined with solid-state polymerization has been used to create a porosity gradient in polyglycolide scaffolds both in the macroporous (>100 µm) as well as in the microscopic (<1 µm) scale [9]. Kathuria et al. used the cryogel technology to synthesize chitosan–gelatin matrix and created pore materials using freeze-drying technology for 3D scaffold [36]. Freeze-drying is one of the popular methods for synthesizing porous polymeric scaffold/matrices, though it is time consuming and an expensive process. It involves two main steps freezing and drying. The principle in freeze-drying is sublimation, the conversion of a solid (ice) directly into its gaseous form (water vapor). Vlierberghe et al. prepared porous gelatin scaffolds by cryogenic treatment of a chemically cross-linked gelatin hydrogel, followed by removal of the ice crystals formed through freeze-drying [37]. Temperature gradients during controlled cryogenic treatment were applied to induce a pore size gradient in gelatin hydrogels. With implementing temperature gradients of 10 and 30 °C during the freezing step, resulting in scaffolds with average pore diameters of, respectively, ±116 and ±330 µm.

Take inspiration from the above research, in the study, a gradient mesh template which can be prepared by the methacrylic anhydride cross-linked gelatin combining with temperature gradient field method. Then, PDMS–E grafted gelatin (PGG) was added into the mixed organic solvents for preparing colloidal particles. Next, the PGG colloidal particles filled into the gradient mesh template. A filling approach for the preparation of gradually distributed films along the thickness of the film was presented. A longitudinal polymer gradient hydrophilic/hydrophobic film was obtained with the Si elemental content exhibiting a typical gradient distribution pattern along the thickness of the film. The gradient film not only overcame the water-resistance and poor mechanical properties of gelatin film, but presented the favorable thermal stability and excellent water vapor permeability. The gradient film has potential application in biomaterials and leather finishes.

## 2. Materials and Methods

### 2.1. Materials

Gelatin (type B, isolated from bovine skin, isoelectric point: 5, Bloom strength: 257), was purchased from China National Medicines Corporation Ltd. and used without further purification or treatment. Methacrylic anhydride (MAA) was obtained from Aladdin Inc. Dialysis membranes (molecular weight cutoff of 10,000 Da) were procured from the US. 1-[4-(2-Hydroxyethoxy)-phenyl]-2-hydroxy-2-methyl-1-propane-1-one (Irgacure^®^ 2959), sodium dodecyl sulfate (SDS), ally glycidyl ether (AGE) and H_2_Pt_6_Cl_6_ catalysis were supplied from Alfa Aesar. Hexamethylcyclotrisiloxane (D3, >95%), *n*-butyllithium (C_4_H_9_Li, 99+%), chlorodimethylsilane (C_2_H_7_ClSi, 99+%) were purchased from Sigma-Aldrich. D3 was put in a flask, diluted with an equal volume of purified benzene, and stirred over CaH_2_. The solvent was distilled and the monomer was sublimed into a flask [38]. Benzene (GR), tetrahydrofuran (THF, GR), ethanol (AR), and acetone (AR) were purchased from Sigma-Aldrich and were dehydrated strictly before used.

### 2.2. Preparation of Gelatin Mesh Template

Gelatin methacrylamide was prepared by reacting gelatin with MAA. The gelatin-MAA (*w*/*v*) ratios were confirmed to be 1.0:0, 1.0:0.06, 1.0:0.12, 1.0:0.18, 1.0:0.24, 1.0:0.30 and 1.0:0.36. First, gelatin was dissolved in phosphate buffer (pH = 7.5) at 50 °C for 1 h. Then, a specific amount of MAA (0, 0.3, 0.6, 0.9, 1.2, 1.5, or 1.8 mL) was added to the above solution with stirring at 50 °C. For gelatin-MAA ratio of 1.0:0, the reaction was continued without the addition of MAA for 2 h. Then the reaction solutions were dialyzed at 40 °C for 24 h to remove the small molecules produced in this process. After the residual solutions were freeze-dried, the gelatin-MAA was dissolved in 30 mL distilled water. Stock solutions of concentration at 3%, 5%, 8% and 10% were obtained and they were then stirred for 2 h at 40 °C. The initiator, Irgacure^®^ 2959 (2 mol %), was added for the cross-linking of gelatin-MAA. The obtained solution was poured into a per-set mold. After standing for 1 h at room temperature, the formed hydrogel was exposed to UV light (254 nm) for 2 h. This process improved the cross-linking between the gelatin and MAA molecules.

**Scheme 1:**
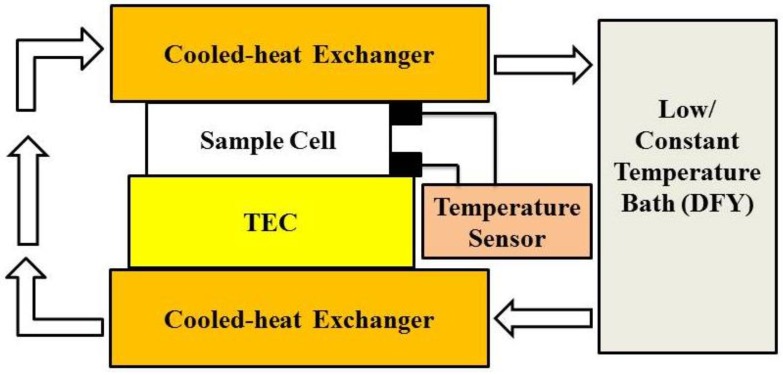
Overview of the preparation of gelatin mesh template.

Then, the cross-linking hydrogel was placed in a self-regulating temperature controller (see Scheme 1). The controller included a low/constant temperature water bath (DFY), a thermoelectric cooler (TEC), two cooled-heat exchangers and a sample cell. The DFY controlled temperature from −25 to 50 °C. The TEC controlled temperature from −30 to 30 °C, and the actual value sensed by temperature sensor. DFY and TEC were simultaneously opened. The temperature of TEC was kept at −20 °C, and the temperature of DFY tuned from −20 to 10 °C. The gradient temperature at 0, 10, 20, 30 °C can be obtained. The hydrogel sample was placed in the center, between TEC and cooled-heat exchanger. A temperature driven gradient was obtained from the high temperature to the low temperature region. After 1 h, the concretionary hydrogel was freeze-dried, and a mesh template was obtained. The pore morphology and porosity of the mesh template was studied by optical microscopy (OM) and Image J.

### 2.3. Preparation of PDMS-E Grafted Gelatin Colloidal Particles

#### 2.3.1. Synthesis of PDMS-H and PDMS-E

**Figure 1:**
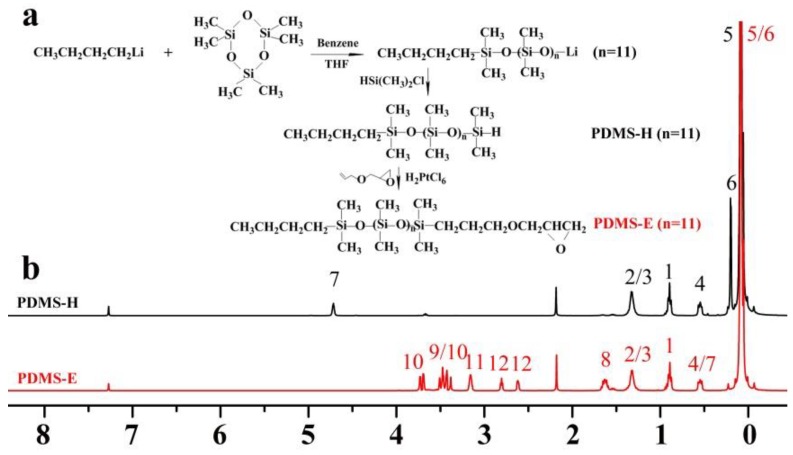
(**a**) Scheme of PDMS-H and PDMS-E polymer synthesis. (**b**) ^1^H NMR spectra of PDMS-H and PDMS-E in CDCl_3_ at 25 °C.

D3, C_4_H_9_Li and C_2_H_7_ClSi were used to synthesize PDMS with Si-H group at one end (PDMS-H) through anionic addition polymerization. First, 10 mL of benzene was added to the flask, and then 24 mL of C_4_H_9_Li was added. After reducing pressure and ventilation with argon gas, 45.99 g of D3, which was first dissolved in 40 mL benzene, was added to the flask. After reaction for 30 min, 50 mL of THF were added into the flask to react for 8 h. Then, 11 mL of C_2_H_7_ClSi were injected into the flask to stop reaction. The obtained PDMS–H products were purified. The outcome of the polymerization reaction was ~85%. Then, purified PDMS-H and ally glycidyl ether (AGE) under H_2_Pt_6_Cl_6_ catalysis were used to synthesize PDMS-E, (*M*_w_ = 1.14 × 10^3^ g·mol^−1^, *M*_w_/*M*_n_ = 1.16). ^1^H nuclear magnetic spectra (^1^H NMR) spectra of PDMS-H and PDMS-E were shown in Figure 1.

#### 2.3.2. General Procedure for PDMS-E Grafted Gelatin Reaction

All the samples were prepared from a stock solution of gelatin in order to minimize the experimental errors. The stock solution was prepared by dissolving gelatin in distilled water (5%, *w*/*w*), and stirred for 3 h. Then the gelatin solution was heated to 50 °C to ensure complete dissolution. SDS (6%, *w*_surfactant_/*w*_gelatin_) was added to the solution with stirring for 6 h. Subsequently, pH of each solution was adjusted to 10.0 using sodium hydroxide (NaOH, 2.0 mol·L^−1^ solution. PDMS-E was added to the gelatin solution at 50 °C at an interval of 20 d·min^−1^ with stirring at fixed PDMS-E/gelatin ratio. PDMS–E was added to the gelatin solution at 50 °C for 20 min under stirring at a predetermined PDMS/gelatin ratio of 0.8:1 (mol:mol). The contents were allowed to react for 24 h. The content of free –NH_2_ groups was evaluated by the Van Slyke method each hour.

#### 2.3.3. Preparation of Colloidal Particles

A mixed solvent system can induce the self-assembly of PGG. In the present work, ethanol/water and acetone/water solvent systems were chosen for inducing self-assembly so as to adjust the size of PGG. The size distributions are summarized in Table S1 and the optical microscopic images of PGG in different solvent systems are shown in Figure S1. Based on the data in Table S1 and Figure S1, it could be concluded that size distributions of PGG in ethanol/water with ratios of 3:1, 9:1 were smaller than that of PGG in acetone/water with a ratio of 3:1.

### 2.4. Porosity Test

In this paper, the conventional image analysis software (Image J, Bethesda, MD, USA) was used to calculate and analyze porosity and pore size distribution in cross section of gradient film based on scanning electron microscopy (SEM) images. Firstly, color SEM images were converted to 8-bit grayscale images which were segmented used. Thirteen separate grayscale values were assigned to the porosity. The pores were traced manually along with the thick direction on the section of gradient film, with a maximum error of ±2 voxels. Then, the parameters of measure were selected in software for ensuring that all the gray level measurements were selected. Finally, the porosity and pore size distribution were calculated by the statistical method. In the study, pore sizes and the volume of porosity of gelatin mesh templates were measured using this software.

### 2.5. Filling Colloidal Particles

First, deionized water (~2 mL) was added to PGG and stirred for 2 h. Subsequently, the mixtures were blended with ethanol or acetone at varying water-to-solvent volume ratios (denoted as X:1 by volume, X = 3, 9). After stirring for 6 h, the mixtures were injected into the gelatin mesh template that was placed in the Brucella funnel, and then sealed and pumped into a vacuum. The filled samples were dried at room temperature for 48 h.

### 2.6. Characterization

#### 2.6.1. Scanning Electron Microscope

The gradient PGG film was cut open along the thickness of the film. Cross sectional micrographs were taken using scanning electron microscope (FEI Co., Hillsboro, OR, USA) secondary electron imaging (SEI) detector. The samples were sputtered by gold for 30 s, and the electricity kept in 60 mA. Frozen section was a conventional method for observation cross-sectional profiles of biomaterials. In this, 8-serial sections were cut along thickness of the filled film at a thickness of ~160 μm (Leica CM1520, Solms, Germany). The pore sizes were calculated by SEM images of the sections and Image J.

#### 2.6.2. Energy Dispersive X-ray

Serial section of the gradient PGG film (~100 µm) was obtained by freezing microtome section technique. EDX analysis was carried out on the opaque surfaces to determine the elements in the sections. The concentrations of C, O and Si elements were collected from eleven serial sections from upper surface to bottom surface to calculate the distribution of the elements.

#### 2.6.3. Fourier Transform Infrared Spectroscopy

FT-IR spectrophotometer (Bruker Tensor-27 FTIR, Karlsruhe, Germany) was used to obtain information about functional groups in the gradient film at different sections. The samples were scanned from 4000 to 400 cm^−1^ with the resolution of 4 cm^−1^.

#### 2.6.4. Contact Angle

The contact angle experiments were operated by A KSV CAM200 goniometer of the samples (KSV Company, Seoul, South Korea). Measurements were performed in a clean-room environment to eliminate the possible impact of air impurities on the water contact angle measurements.

#### 2.6.5. Thermal Gravimetry Analysis (TGA)

Thermal gravimetric analysis was carried out to observe changes in thermal events in the paint films using Shimadzu TGA-51 (Tokyo, Japan). The paint films and the pure copolymer sample were heated from 25 to 1000 °C and 25 to 600 °C at a rate of 10 °C/min under 40 cm^3^/min nitrogen flow, respectively.

#### 2.6.6. Differential Scanning Calorimetry (DSC)

The thermal properties of the gelatin films were measured using a Perkin Elmer Pyris Diamond DSC at a heating rate of 10 °C/min under nitrogen atmosphere. The temperature and enthalpy scales were calibrated using standard samples of indium and zinc. The gelatin sample was sealed in a hermetic pan to prevent any loss of moisture during DSC measurement.

#### 2.6.7. Dynamic Mechanical Analysis

DMA of the samples were obtained from gradient, non-gradient or gelatin films, respectively. The length of these films was all 5.0 cm with the wide of 1.0 cm and thickness of 2.0~2.1 mm. These films were prepared by drying at room temperature, and then placed in environment with constant temperature (25 ± 1 °C) and humidity (30 ± 2%) for 48 h. The measurements were carried out on a Perkin Elmer PYRIS Diamond DMA (PerkinElmer, Inc, Waltham, MA, USA) in the tensile mode at a frequency of 1 Hz. The temperature range was set from −5 to 260 °C, with a heating rate of 5 °C/min.

#### 2.6.8. Particle Size Analysis

The emulsion particle size distribution was obtained by Zetasizer 3000 (Malven Instruments, Malvin, UK). Firstly, the PGG colloidal liquid was put into a color matching test tube carefully. Then, the tube was put into the ZetaSizer 3000 laser particle instrument to measure the particle size distribution.

#### 2.6.9. Testing of Primary Amino Conversion Rate

Primary amino group content is measured by a modified Van Slyke method and the primary amino conversion is calculated [39]. The weight of each sample of the reacted gelatin solution was weighed approximately 7.760 g, the measurement temperature was 50 °C, and 45 min. The conversion of the primary amino groups in the grafting reaction was calculated from the change in the numerical value before and after grafting of the primary amine groups on the collagen polypeptide molecular chain. The specific calculation equation was as follows:

$$P=\frac{{{[\mathrm{NH}}_{2}]}_{0}-{{[\mathrm{NH}}_{2}]}_{t}}{{\left[{\mathrm{NH}}_{2}\right]}_{0}}\times 100\%$$

P is the conversion of the primary amine group of the grafting reaction; [NH_2_]_0_ is the primary amine group content before the grafting (mol/g); [NH_2_]_t_ is the primary amine group content after the grafting reaction (mol/g).

#### 2.6.10. Water Vapor Permeability Studies

**Scheme 2:**
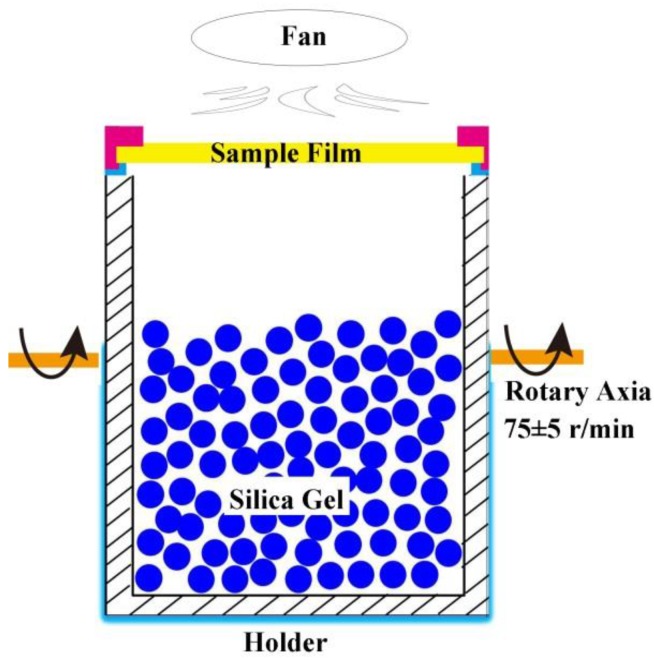
Schematic diagram of the water–vapor transmission device.

In the study, dynamic water vapor permeability (dWVP) was used. The method of dWVP is based on the measurement of China Light Industry Standard QB/T1811-1993. The apparatus was done by GJ9E1 water vapor transmission rate instrument (Co. light industrial machinery, Hefei, China). Permeability studies were performed in an apparatus consisting of three main parts, including test-bottle, holder and fan, as shown in Scheme 2. Test-bottle (bottleneck diameter 30 mm) was the bottom part which included silica gel (59 ± 0.5 g,). The silica gel was standing on dryer for 6 h when it was dried at 125 ± 5 °C oven for 16 h. The test-bottle was supported by a holder. The holder can be driven by electrical machinery, and the rotate speed was 75 ± 5 r/min. The third part is a fan which was right above the bottleneck. The fan conveyed moist air getting through the sample. The testing film was fitting smoothly into the bottleneck through mechanical operation. The temperature of experiment room was 20 °C. During typical dynamic permeation experiments, the relative humidity of permeable air was 65 ± 2%. After 16 h, the sample absorbed the moist air was moved quickly to another bath including silica gel. The weight of silica gel was firstly tested by balance (0.001 g, named as m_1_). After 7 h, the experiment was finished. The weight of silica gel was further tested by balance (0.0001 g, named as m_2_). The difference between m_2_ and m_1_ was recorded. Based the Standard, WVP (mg/cm^2^·h) was calculated as follows:

WVP = 7639ΔW/St.

where ΔW (mg) is the weight gain of the testing film; S (cm^2^) is the area of the exposed film (bottleneck area); t (h) is the time during which a steady state occurred. Two replicates of each film were tested.

## 3. Results and Discussion

### 3.1. Pore Structure of Gelatin Mesh Template

Porosity and pore sizes were extremely important in understanding the structure of gelatin mesh template [37]. Pore sizes obtained from the SEM images and porosity analyzed by Image J. We found that there were many factors affecting the calculated results, including conversion of primary amino groups in the synthesis of gelatin-MAA, concentration of gelatin-MAA, and temperature gradient.

**Figure 2:**
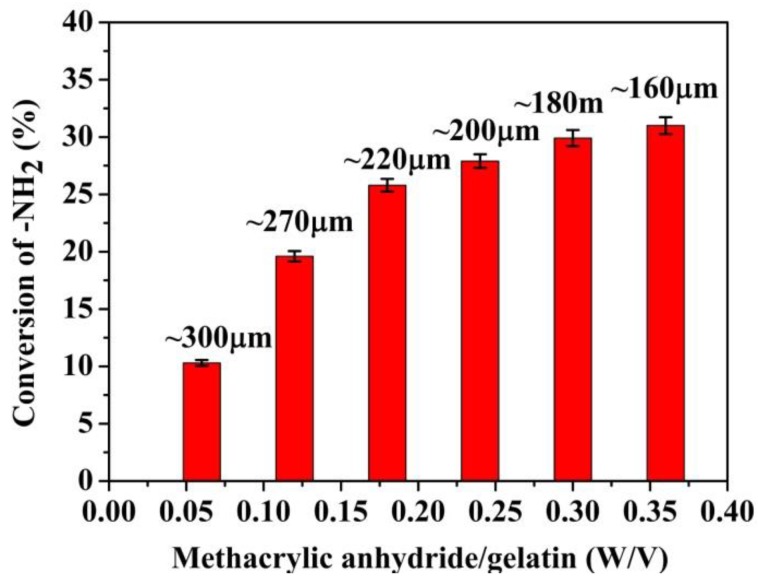
The effect of MAA to gelatin ratio on the pore size. SD 0.3, *n* = 3.

The result of Figure 2 indicates that when gelatin/MAA (*w*/*v*) ratio increased from 1.0:0, 1.0:0.06, 1.0:0.12, 1.0:0.18, 1.0:0.24, 1.0:0.30 to 1.0:0.36, conversion of primary amino groups increased correspondingly from 0 to 31%, but the pore size decreased from ~300 to ~160 µm. The initiator facilitated the formation of covalent bonds between gelatin and methacrylamide molecules. Crosslinking degree increased as the conversion of –NH_2_ increased, along with the formation of more and more dense pores. The mesh template with dense pores is beneficial to prevent collapse during the filling of colloidal particles [40]. Gelatin-MAA with different conversion rates of –NH_2_ was used for the preparation of the gradient mesh, keeping the concentrations of other components constant. This made is possible to probe the effects of conversion of –NH_2_ groups on the formation of the gradient mesh. Results indicated that high conversion of –NH_2_ groups (~31%) was beneficial for the formation of optimum pore sizes of the gradient mesh.

**Figure 3:**
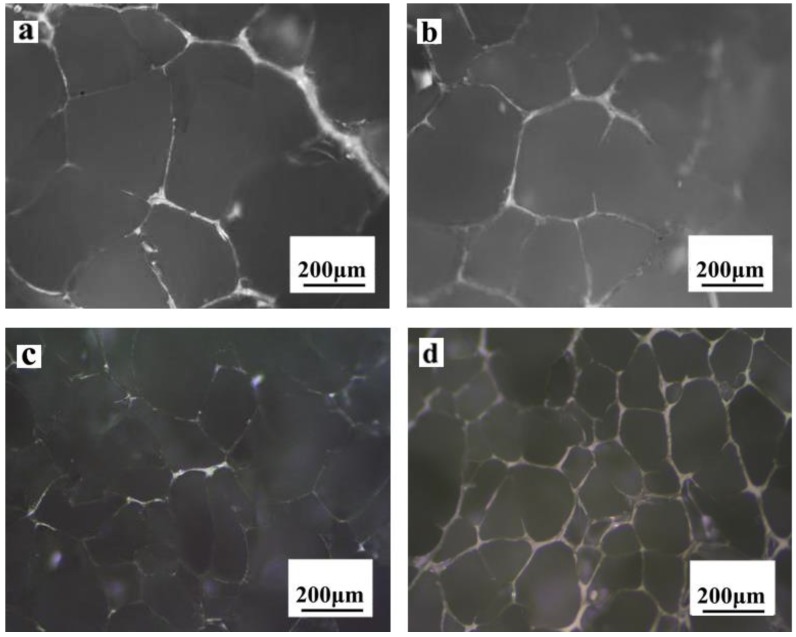
The effect of (**a**) 3%, (**b**) 5%, (**c**) 8% and (**d**) 10% concentration of gelatin methacrylamide on the pore size.

The results of Figure 3a–d showed that the concentration of gelatin-MAA importantly affected the pore structure of template. With the concentration of gelatin-MAA increasing from 3 to 10% (*w*/*v*), pore size decreased from ~400 to ~130 µm. This ascribed that the more concentrated hydrogels resulted in smaller pores [37].

**Figure 4:**
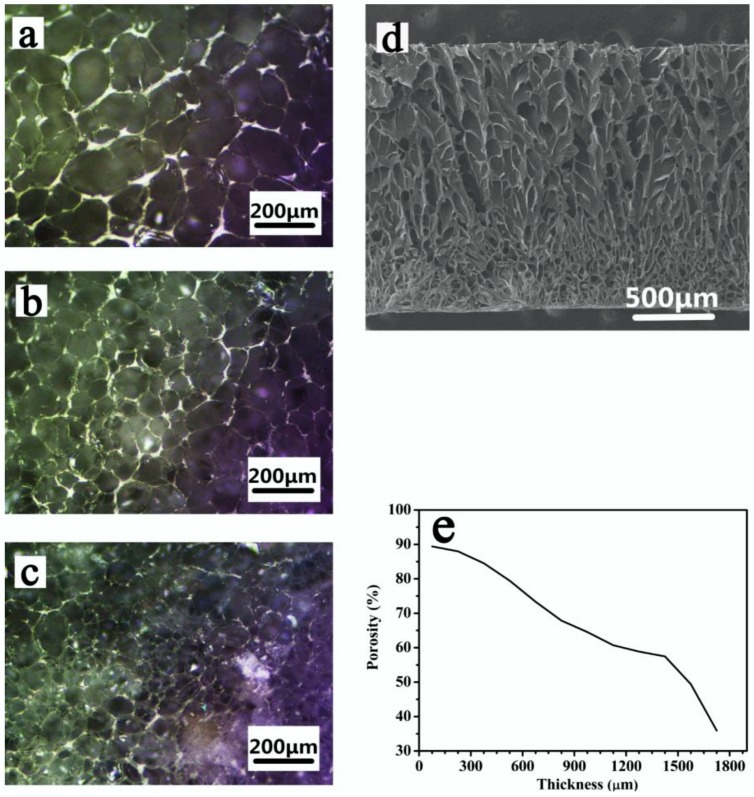
Pore morphologies of the gelatin substrate. (**a**) At the top; (**b**) in the interlayers; (**c**) at the bottom; (**d**) the vertical section; (**e**) porosity of gelatin substrate from top to bottom.

The temperature gradient also played a key role in determining the pore size. Tuning ∆*T* = 0, 10, 20 and 30 °C, the larger variations in temperature from TEC (see Scheme 1) to cooled-heat exchanger resulted in appropriate variations of pore sizes along the thickness of the film. Figure 4a–c clearly shows the pore morphologies of the slices in top, center and bottom parts. The average pore size in the top part was largest (~160 µm), and those in the center and in the bottom part were about 130 µm and 50 µm, respectively. The topmost was ~240 µm, and the underside (exposed to the lowest temperature) was tight network in which smaller pores were observed (3~5 µm). Figure 4d shows the intact sectional view by SEM. It was observed that the continuous space network porous structure of the section was formed, which indicated that the pore size gradually decreased from the top to the bottom. Figure 4e and Table S2 presents the gradation in porosity which was calculated by Image J program [9]. It was evident that the porosity decreased gradually with decreasing pore size. Similar results were also reported by Vlierberghe [38]. Based on these results, the optimal conditions were found to be –NH_2_ conversion (~30%), the concentration of gelatin-MAA (10%) and ∆*T* (30 °C). Under these conditions, a proper gelatin mesh template was obtained. This result provided an important clue that the size of the colloidal particles was an important condition for their filling or the preparation of the gradient film.

### 3.2. Filling of Colloidal Particles

**Figure 5:**
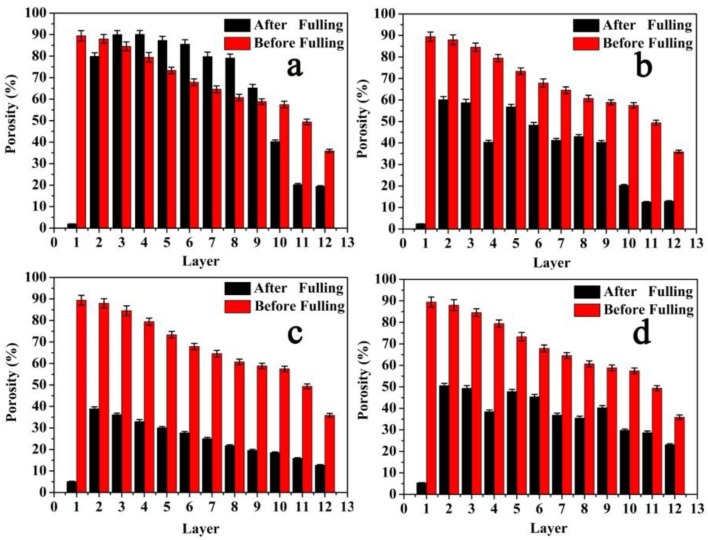
Filling rates of PGG in different solvent mixtures. (**a**) ethanol/water at 0:1; (**b**) ethanol/water at 3:1; (**c**) ethanol/water at 9:1; (**d**) acetone/water at 3:1. SD 0.3, *n* = 3.

In the filling process, the sizes of PGG colloidal particles, vacuum, and drying methods determined the filling rates and concentration gradient of PDMS components along its thickness. PGG can aggregate in mixed solvents. In this work, ethanol/water and acetone/water solvent systems were chosen to induce aggregate and adjust the size of PGG. As shown in Table S1 and Figure S1, when PGG was mixed in ethanol/water solvent system in ratios of 0:1, 3:1, and 9:1 and in 3:1 acetone/water mixture, the size distribution of PGG was 1~24, 1.2~13, 0.9~10 and 1~25 µm, respectively. The size distribution of PGG in 9:1 (*v*/*v*) ethanol/water was lower than other conditions. The four kinds of PGG aggregates were filled in the gelatin mesh template. After freezing with liquid nitrogen, the sample was cut across with a blade following the direction of temperature gradient for characterizations. The variations in porosity across the thickness were showed in Figure 5a–d. Figure 5c showed that there was a gradation in the filling rates. Moreover, PGG colloidal particles were evenly piled up, which prevented the collapse of the gelatin mesh. These results implied that the smaller size of PGG particles was easily filled into the pores. However, the colloidal particles did not completely fill with the gradient template. Four vacuum conditions (0, 0.02, 0.04, 0.06, 0.08 MPa) were chosen to tune the filling rates of PGG aggregates. A higher vacuum led to the collapse of the gelatin mesh template, and a lower vacuum resulted in incomplete filling of the pores. Vacuum of 0.06 MPa led to better filling of pores. Last, the effects of drying at room temperature or at 40 °C were studied. As the temperature increases to 40 °C, gelatin undergoes transformation from solid to gel, and then sol phases, which has an adverse effect on the filling of samples to retain their original state. However, at room temperature, physical state (solid) of gelatin has been kept, which has no obvious effect on the structure of film. A satisfied result was obtained when drying was done at room temperature. Hence, the optimal conditions for filling were as follows: PGG aggregates in 9:1 (*v*/*v*) ethanol/water, 0.06 MPa vacuum, and drying at room temperature.

### 3.3. Hydrophobicity/Hydrophilicity

**Figure 6:**
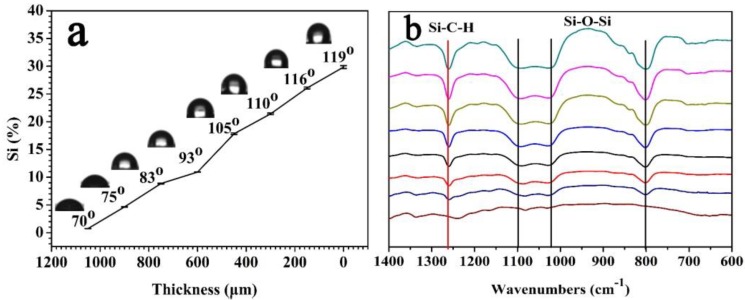
(**a**) Concentration of Si element in the slices along the vertical direction from top to bottom by SEM–EDS and corresponding contact angles of the slices. SD 0.2, *n* = 3. (**b**) FT–IR spectra of the slices (top to bottom).

The optimal film was laid on the cooling stage (−25 °C) and covered by water, then, the freezing films were microtomed. The thin sections were ~110 μm in thickness. Eight slices were obtained from the optimal film (thickness ~1100 μm) using the freezing microtome. These slices were characterized by elemental mapping using SEM-EDS, FT-IR spectroscopy, contact angle measurements, and DMA. Results of elemental mapping by SEM-EDS were as shown in Figure 6a, abscissa is on behalf of the thickness, and ordinate shows the change of the concentration of Si element along the thickness. A typical gradient distribution of Si element along the thickness was presented. Figure 6b showed that the contact angle of every slice decreased with decreasing Si content. These results clearly indicated that hydrophobicity gradually decreased with decreasing PDMS concentration along the thickness. FT-IR spectra showed peaks at 790 and 1262 cm^−1^, due to Si–CH_3_ groups and out-of-plane vacillation vibrations of the C–H bond, respectively. Moreover, the absorptions between 1100 and 1001 cm^−1^ could be attributed to the vibrations of Si–O bonds in Si–O–Si structure [10]. These characteristic absorption peaks deceased gradually with decrease in PDMS concentration along its thickness. This suggested that PDMS was gradually distributed along the thickness of the film. Hence, PGG compositional gradient film was successfully fabricated.

### 3.4. Thermal and Mechanical Properties

**Figure 7:**
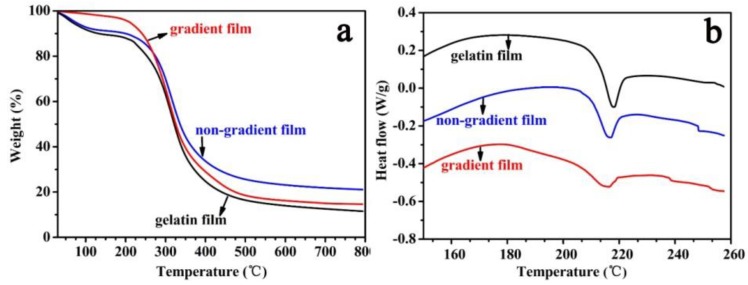
(**a**) TGA and (**b**) DSC curves of gradient, non-gradient and gelatin films.

TGA is a standard technique to evaluate the thermal stability of materials. TGA results showed only one plateau region for gradient film, whereas non-gradient film (ungraded film) and gelatin film showed two plateau regions (Figure 7a). The mass loss of about 8% before 100 °C (first plateau region) is assigned to the evaporation of the water in the non-gradient and gelatin films. When the temperature is over 250 °C, there is a rapid weight loss (second plateau region), which comes from the thermal degradation of the gelatin. No further large mass losses are observed when the temperature is above 400 °C. The results reflected that thermostability of gradient film was obviously enhanced for gradient film, which was ascribed that the hydrophobicity of PDMS chains hindered the water absorption, and reflected that the stacking of the polymeric chains in the gradient film was different than in other films [41].

DSC results showed that the *T*_g_ of gelatin film, gradient film and non-gradient film were about 218, 215 and 217 °C (Figure 7b), respectively, which indicated that the three kinds of films had similar *T*_g_. However, enthalpy relaxation strength of the gradient films obviously decreased. The result indicated the gradient structure was different from random structure of pure gelatin and non-gradient films. This result suggested that the mobility of the polymeric chains increased relatively at low temperatures, and the flexibility of the gradient film increased to a certain extent.

**Figure 8:**
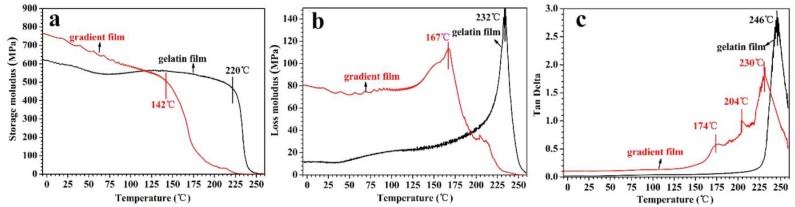
(**a**) Storage-moduli, (**b**) loss-moduli, and (**c**) tan delta values of the gradient and gelatin films, measured by DMA in the tensile mode.

DMA results are shown in Figure 8. Storage-moduli and loss-moduli values indicated that the temperature inflection point of the gradient film was lower than that of the gelatin film. The gradient film of energy storage modulus and loss modulus at low temperature is larger than that of gelatin film, which shows that the viscoelasticity of the gradient film was better compared with the pure gelatin film. However, at high temperature the result was opposite. Hence, polymer chains could move more freely in the gradient film at low temperature. This could be attributed to the excellent flexibility of the PDMS chains. These results implied that the flexibility of gradient film was improved. In addition, the results of storage-moduli and loss-moduli of the slices in the top, central, and bottom regions also indicated that flexibility decreased with the decrease of PDMS concentration (Figure S2). Tan delta values indicated that phase-separated structures existed in the gradient film, which was the cause for increased flexibility of the chains in the gradient film.

### 3.5. Water Vapor Permeability

To determine the effect of gradient structure on the barrier property of the gelatin films, water vapor permeation (WVP) experiments were conducted. The permeabilities were estimated and the data are listed in Table 1. Gelatin is natural macromolecules which obtained from skin, bone or tendon. Excellent water vapor permeability is intrinsic characteristics of gelatin materials. The grafting of PDMS-E obviously hindered the water vapor permeation for modified gelatin film. However, gelatin film with gradient structure obviously surpassed that with disordered structure. Maybe, the size, shape and the geometry of the gradient structure results in the difference.

## 4. Conclusions

Gradient films were prepared by incorporating PDMS components into a gradient gelatin mesh template. The distribution of PDMS was gradual along the thickness of the film. This was done by filling the colloidal particles of PGG with a suitable size into the gradient mesh template. The porosity of the template decreased from 89 to 35%. This was accompanied by a decrease in average pore size from 160 to 50 µm. Colloidal particles of about 0.9~10 µm were formed from PGG after adding them to a mixed solvent system of 9:1 (*v*/*v*) of ethanol and water. This was filled in the mesh template under vacuum (0.06 MPa), and after drying at room temperature for 48 h, a gradient film was obtained. SEM-EDS combined with freezing microtome and FT-IR results suggested that the distribution of the Si element along the thickness showed a typical gradient pattern, which led to hydrophilic/hydrophobic continues changing along the thickness of film. The water vapor permeability, TGA, DSC, and DMA results showed that the gradient films had excellent water vapor permeability and flexibility, and hence could be used as biomimetic materials and leather finishing agents.

## Figures and Tables

**Table 1:** Water vapor permeabilities of the gradient, non-gradient and gelatin films.

Sample	Gelatin Film	Gradient Film	Non-Gradient Film
Water vapor permeability (mg/cm^2^·h)	1288.1 ± 5 1349.6 ± 5	1093.4 ± 5 1078.3 ± 5	608.3 ± 5 612.4 ± 5

## Supplementary Materials

The following are available online at http://www.mdpi.com/2073-4360/10/6/677/s1, Table S1: Size distributions of PGG in ethanol/water at 0:1, 3:1, 9:1 and acetone/water at 3:1, Table S2: the statistical data of Image J, Figure S1: Optical microscopic images of PGG in ethanol/water ratios of (a) 0:1, (b) 3:1, (c) 9:1 and (d) acetone/water ratio of 3:1,Figure S2: Store-moduli and loss-moduli of the top interlayer, and bottom sections, as measured by DMA in the tensile mode.
