# Supplementary material for: Preparation of Compositional Gradient Polymeric Films Based on Gradient Mesh Template
Source: Polymers (Basel). 2018 Jun 18;10(6):677. doi: 10.3390/polym10060677 (PMC6404138; doi:10.3390/polym10060677)
Supplement: Supplementary file 1 [file polymers-10-00677-s001.zip › polymers-312128-supplementary.docx]

**Supporting Information**

Preparation of Compositional Gradient Polymeric Materials Based on Gradient Mesh Template

Honglei Teng ^1^, Jing Li ^1^, Zhaosheng Hou ^2^, Xilu Yan^1^, Rulin Han^1^, Jing Xu^1,^* and Tianduo Li^1,^*

^1^ Shandong Provincial Key Laboratory of Molecular Engineering, School of Chemistry and Pharmaceutical Engineering, Qilu University of Technology(Shandong Academy of Sciences), Jinan, Shandong, 250353, China. Tenghonglei1122@163.com (H.T.); lijing@163.com (L.J.);

^2^ College of Chemistry, Chemical Engineering and Materials Science, Shandong Normal University, Jinan, Shandong, 250100, China. houzs@sdnu.edu.cn (Z.H.)

***** Correspondence: xujing@qlu.edu.cn, litianduo@163.com

Received: date; Accepted: date; Published: date

1. *Characterization of the Size of PGG*

A mixed solvent system can induce the self–assembly of PGG. In the present work, ethanol/water and acetone/water solvent systems were chosen for inducing self–assembly so as to adjust the size of PGG. The size distributions are summarized in Table 1 and the optical microscopic images of PGG in different solvent systems are shown in Figure S1. Based on the data in Table 1 and Figure S1, it could be concluded that size distributions of PGG in ethanol/water with ratios of 3:1, 9:1 were smaller than that of PGG in acetone/water with a ratio of 3:1.

Table S1. Size distributions of PGG in ethanol/water at 0:1, 3:1, 9:1 and acetone/water at 3:1

| solvent | size distribution (µm) |
| --- | --- |
| ethanol/water (0:1)  ethanol/water (3:1)  ethanol/water (9:1)  acetone/water (3:1) | 1~24  1.2~13  0.9~10  1~25 |


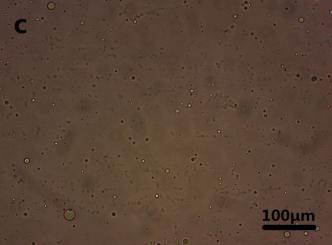

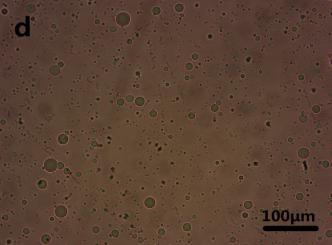

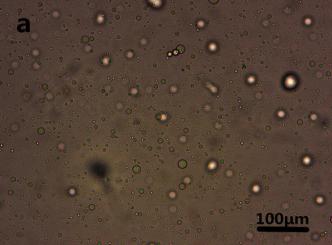

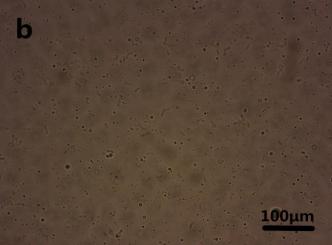


**Figure S1.** Optical microscopic images of PGG in ethanol/water ratios of **(a)** 0:1, **(b)** 3:1, **(c)** 9:1 and **(d)** acetone/water ratio of 3:1.

1. *Pore analysis for Figure 4d*

In this paper, the conventional image analysis software (Image J, Bethesda, MD) was used to calculate and analyze porosity and pore size distribution in cross section of gradient film based on scanning electron microscopy (SEM) images. The specific operation steps were as follows:

**Open the picture**: File->Open->Image to be analyzed.

**Grayscale conversion**: click Image->Type->8-bit grayscale. Color SEM images were converted to 8-bit grayscale images*.* The images were segmented using. Thirteen separate gray scale values were assigned to the porosity. Manually track holes in the thickness direction.

**Select the threshold**: click, Image->Adjust->Threshold, click on “Set” to set the threshold of the image. The range of threshold was 0~255.

**Select “Analyze–Set Measurements” and choose the parameters to be measured**. The parameters included background color (contrasting with the color of the hole) and the range of pore size (1μm~∞) for ensuring that all the gray level measurements were selected.

**Select“Analyze–Measure”/ “Analyze–Analyze Particles”**. Outlines, Display Results and Exclue on Edges were carried out.

**Measurement**: Use the statistical method to calculate the porosity [1,2]. The error is ±2 pixels.

The statistical data were shown in Table S2.

Table S2. Porosity statistics of gelatin substrate from top to bottom.

| Depth/μm | Porosity/% | Error/% |
| --- | --- | --- |
| 75 | 89.43 | 0.1905 |
| 225 | 87.99 | 0.2138 |
| 375 | 84.52 | 0.1826 |
| 525 | 79.45 | 0.2121 |
| 675 | 73.34 | 0.1709 |
| 825 | 67.88 | 0.1649 |
| 975 | 64.54 | 0.1568 |
| 1125 | 60.72 | 0.1491 |
| 1275 | 58.86 | 0.1353 |
| 1425 | 57.47 | 0.1329 |
| 1575 | 49.39 | 0.1302 |
| 1725 | 35.9 | 0.1005 |

1. *DMA of Gradient Material of Top layer, Interlayer and Bottom layer*

The gradient material was sliced into three sections from the top to bottom, and the mechanical performances of three slices were analyzed by DMA. DMA analyses showed that the mechanical properties had a typical gradation along the thickness of the material, as shown in Figure S2.


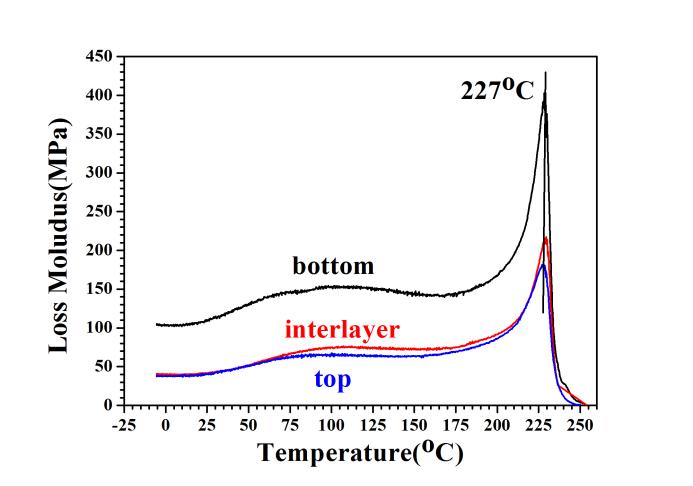

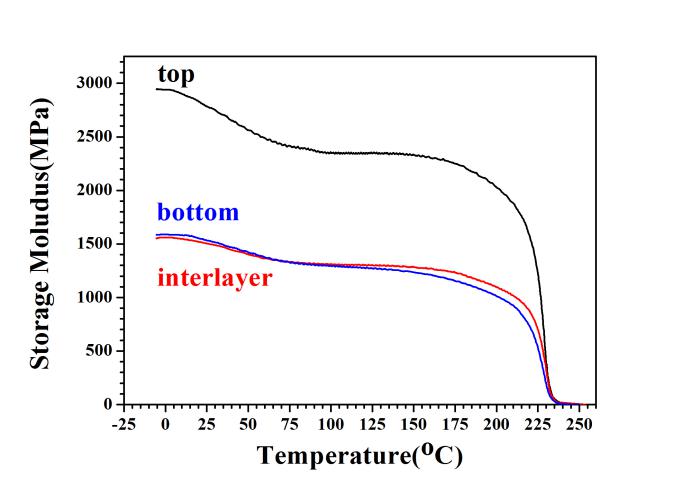


**Figure S2.** Storage–moduli and loss–moduli of the top, interlayer, and bottom sections, as measured by DMA in the tensile mode.

References

### [1]  Jensen, E.C. [Quantitative analysis of histological staining and fluorescence using ImageJ](http://onlinelibrary.wiley.com/doi/10.1002/ar.22641/full). The anatomical record 2013, *296*, 378–381.

[2] The ImageJ User Guide 1.44 Tiago Ferreira • Wayne Rasband. February **2011**, *9*.
